# Effect of teaching health-promoting behaviors on the care burden of family caregivers of hemodialysis patients: a four-group clinical trial

**DOI:** 10.1186/s12912-023-01604-2

**Published:** 2023-11-17

**Authors:** Mehrdad Hayati, Razieh Bagherzadeh, Mehdi Mahmudpour, Fatemeh Heidari, Hakimeh Vahedparast

**Affiliations:** 1 Department of Nursing, Nursing and Midwifery Faculty, Bushehr University of Medical Sciences, Bushehr, Iran; 2 Department of Midwifery, Nursing and Midwifery Faculty, Bushehr University of Medical Sciences, Bushehr, Iran; 3 The Persian Gulf Tropical Medicine Research Center, The Persian Gulf Biomedical Sciences Research Institute, Bushehr University of Medical Sciences, Bushehr, Iran

**Keywords:** Chronic Kidney Disease, Health-promoting behaviors, Caregiver, Care burden, Hemodialysis, Teach-back method

## Abstract

**Introduction:**

Chronic kidney disease could have a profound effect on the life of patients and family caregivers. The caregivers’ care burden increases as the disease progresses. Interventions reducing care burden should be investigated. Educational interventions could affect family caregivers’ care burden among hemodialysis patients. However, most studies and interventions have focused on caregivers. Therefore, this study aims to compare the effect of teaching Health-promoting behaviors on the care burden of family caregivers of hemodialysis patients.

**Materials and methods:**

This trial was conducted using a pretest-posttest design and follow-up after one month. Hemodialysis patients and their family caregivers were selected using convenience sampling method. In total, 124 patient-caregiver pairs were divided into four groups of patient-centered education, caregiver-centered education, Patient and caregiver education and control by block randomization (15 blocks of 8 members and 1 block of 4 members) (n = 31 pairs per group). The intervention (teaching health-promoting behaviors) was performed in 8 sessions using the teach-back method, except for the control. The data were collected by patient and caregiver demographic forms and Novak and Guest care burden inventory as well as following the treatment regimen in three stages (before, immediately after and one month after the intervention). Demographic variables were compared among the four groups using ANOVA, Kruskal-Wallis and Chi-square test. The intragroup comparison of the main variables was made using the repeated measures ANOVA with modified LSD post hoc test. The intergroup comparison was made by one-way ANOVA with LSD post hoc test.

**Results:**

Out of 124 caregivers participating in the study, 68 (54.8%) were female. Also, out of 124 patients participating in the study, 86 (69.4%) were male. The mean age of the caregivers and patients was 39.2 ± 11.31 and 54.23 ± 14.20 years old, respectively. There was a statistically significant difference in the mean total care burden scores of the pre-test and post-test between the four groups (p < 0.001). The total care burden decreased in patient-centered, caregiver-centered and Patient and caregiver education groups. However, this reduction in the caregiver-centered and Patient and caregiver education groups was significantly higher than the patient-centered education group (p < 0.001).

**Conclusion:**

The results revealed teaching health-promoting behaviors reduced care burden. Moreover, caregiver-centered approach could reduce care burden more than patient-centered approach. Therefore, this could be used as a supportive method to improve the health of patients and caregivers.

**Trial registration number (TRN):**

IRCT20090522001930N4.

**Date of registration:**

2021-11-12.

## Introduction

The increased number of chronic patients increases their care needs. A significant part of chronic patients’ medical care needs is covered by informal caregivers such as family caregivers during their referral or hospitalization in healthcare centers or at home [1]. End-Stage Kidney Disease (ESKD) is considered a chronic disease, which has affected more than 10% of the general population worldwide [2, 3], so that more than half of this population live in Asia [4]. The prevalence of ESKD is estimated to be nearly 12% among Iranian adults [5]. Hemodialysis is the most common lifelong treatment method for treating this disease [2, 3], which changes the patient’s lifestyle, health status and social roles. These factors could reduce the patient’s living standards in the long run and make them need others’ care and support [6]. Hemodialysis causes physical, mental and social challenges for patients and their caregivers [3].

Hemodialysis is considered a family conflict. Apart from patients, the family caregiver is the person who is greatly affected by ESKD process and treatment [7]. Family caregiver refers to a family member who is in contact with patients and takes care of them. In other words, caregivers solve patients’ treatment and care needs in their daily life and are considered the main source of psychological and emotional support for patients [8, 9]. ESKD patients’ family members often experience stress, depression, marital dissatisfaction, low quality of life, caregiving fatigue, health deterioration and socioeconomic damage due to being involved in the practical aspect of care (commuting to the hemodialysis center, patient symptom management, etc.) and providing psychological support during the patient care process [8, 10, 11], so that family caregivers of hemodialysis patients are referred to as hidden patients because their needs are often ignored and not prioritized [8, 12].

Approximately 349 million people, including those with ESKD, are dependent on their caregivers, so that they provide up to 90% of the patients’ long-term care [9, 13]. Home care quality is closely associated with patient health. However, many family caregivers are untrained and not adequately prepared to perform caregiving tasks, such as providing wound care and monitoring complex medication management [14]. Unlike developed countries, the process of caring for hemodialysis patients is very challenging in low- and middle-income countries, such that family caregivers reported a care burden of more than 20 h per week [8]. Care burden refers to the physical, mental and social anxiety that occurs as a result of caring for chronic patients [6]. Studies have revealed moderate to severe care burden among family caregivers of hemodialysis patients [9, 13].

Considering that caregivers’ care burden increases with disease progress, a solution should be found to reduce care burden [2]. Ni et al. (2022) found that good family functioning could increase family caregivers’ ability to provide practical support for medication adherence among patients with artificial heart valves [15]. Hekmatpou et al. found that teaching patient care to family caregivers reduced care burden and improved life quality of family caregivers of stroke patients [16].

Based on Bodenmann’s (2008) Dyadic Coping conceptual model, there is a synergistic effect between the patient and caregiver, which could be considered an aspect of disease management. Bodenmann stated that chronic disease could affect patients and their caregivers, so that they both could contribute to disease consequences or management [17]. It seems that education and rehabilitation programs are among the best ways to reduce care burden and strengthen adherence to health-promoting behaviors among family caregivers [18]. Health-promoting behaviors refer to those behaviors that enable people to improve their health and that of their society. These behaviors are of particular importance because they could prevent disease complications, reduce pathogenicity, improve quality of life, maintain individuals’ function and independence and reduce care burden [19]. However, this question that “which one could be more effective in reducing the care burden, teaching health-promoting behaviors to patients or family caregivers” remains unanswered.

Nurses play a key role in educating patients and their caregivers. Due to the frequent interaction between nurses and patients and their families, nurses play a crucial role in communicating with patients’ families, and they can provide the knowledge, skills and support needed to improve the quality of home care. Nurses can play an important role in improving the quality of care [20] and reducing the caregiver burden of hemodialysis patients [2, 10] by assessing family health needs and providing accurate and timely education [20]. Based on the Dyadic Coping theory, if patients undergoing hemodialysis or their caregivers receive training related to health-promoting behaviors, it could affect caregivers’ care burden. However, most studies and interventions have focused on caregivers [9, 10]. The mutual effect that caregivers and patients could have on the care burden has been neglected. Moreover, being holistic and comprehensive in nursing shows the necessity of paying attention to all aspects of health and family in the management of chronic diseases and their consequences. Therefore, the present study was conducted to compare the effect of teaching health-promoting behaviors on the care burden of family caregivers of hemodialysis patients.

## Methods

### Design

This four-group parallel randomized controlled clinical trial was conducted to compare effect of teaching health-promoting behaviors on the care burden of family caregivers of hemodialysis patients. These four groups included patient-centered education, caregiver-centered education, patient and caregiver centered education and control. All steps of the study have been done according to Declaration of Helsinki and the instructions of the Ethics Committee of Bushehr University of Medical Sciences (IR.BPUMS.REC.1400.070) and Iranian Registry of Clinical Trials (IRCT20090522001930N4) in a single-blind manner. In this Study, informed consent to participate obtained from participants.

### Patient population and sampling

The participants included patients referring to hemodialysis centers in two big cities of the province and their family caregivers. According to the study by Farahani et al. [2] (2016) and considering the effect size of 1.6, type I error of 0.01 and power of 95%, the sample size was calculated as 16 pairs (16 patients and 16 caregivers) for each group using G power 3.1.9.2. Since this estimated size was for comparing two groups, the sample size was corrected using the sample size correction formula for more than two groups. Finally, the sample size was calculated as 28 pairs considering the withdrawal rate of 10% (n = 31 per group; in total, 124 pairs). The participants (patient-caregiver pairs) were selected by convenience sampling method considering the inclusion criteria. Then, the participants were allocated to four groups by block randomization in order to match the groups and minimize the role of confounding variables. In total, 15 blocks of 8 members and 1 block of 4 members were considered. Random allocation software was employed for blocking [21].

### Inclusion and exclusion criteria

The patient inclusion criteria were undergoing hemodialysis for at least six months, being 18–70 years old, having consent to participate in the study and being at least able to read and write. The patient exclusion criteria were dying during the intervention, suffering from known cognitive and mental disorders (based on the patient’s file) and temporary need for hemodialysis (poisoning, acute kidney failure and guest). The caregiver inclusion criteria were being over 18 years old, being the patient’s main caregiver (based on the patient self-report), having consent to participate in the study and being at least able to read and write. The caregiver exclusion criteria were suffering from known neurological and mental disorders (based on doctor’s diagnosis) and withdrawal of one of the pairs from the study.

### Intervention

The study started after receiving the ethical code and necessary permits. The research objectives and methodology were explained to the participants who met the inclusion criteria. It was emphasized that participating in the study would be voluntary and the participants could withdraw from the study whenever they wish. Then, the informed consent form was completed and the study began. In the four groups of patient-centered education, caregiver-centered education, Patient and caregiver education and control, demographic forms were completed by the patients and caregivers. The care burden inventory was completed by the main caregivers. After completing the questionnaires, the educational intervention began.

The intervention was carried out in one of the rooms of the hemodialysis sector, which was specific for training without the presence of other people. The patient-centered and patient-caregiver groups received training before the hemodialysis. The caregiver-centered group received intervention during the hemodialysis. Teaching was provided to all the participants by a trained nursing expert in the field of health-promoting behaviors among hemodialysis patients. The educational content was prepared based on reliable scientific sources. The opinions of experts, including a nephrologist and two doctors of nursing practice and health education, were used to confirm its content validity. Table 1 presents the educational content. Based on the compiled content, teaching was provided in eight sessions twice a week and duration of each session was 30 min on average, i.e., it varied from 15 to 60 min depending on the patient’s fatigue and desire. Teach-back method was used to teach health-promoting behaviors [22]. This method included five steps: Pre-test, goal setting, implementing teaching process, evaluating and making a decision whether to repeat the above steps based on the patient’s learning and educational goals [23]. The researcher determined patients’ educational needs in the pre-test phase of the teach-back method. This phase lasted 15–60 min. Based on the need’s assessment, cognitive behavioral goals were determined in each session. In the stage of implementing the teach-back method, the process of patient-centered education was implemented. After teaching, feedback was obtained from the participants using open-ended questions based on the needs of each patient (evaluation). In the decision-making stage, the educational content that the patients did not learn properly was repeated in each session until they achieved the educational goal and learned the desired content. At the beginning of each session before starting teaching, the participants were asked to recount the content using open-ended questions based on the teach-back method. If they failed to retell the content properly, the trainer repeated the content focusing on the key points until they fully learn. The control group received no intervention. After the final assessment, the control group received training in order to comply with the ethics in the research. The data were collected three times: Before, immediately after and four weeks after the intervention.

**Table 1:** Comparing frequency distribution of nominal and ordinal demographic variables of caregivers between four groups

		Patient-centered education	Caregiver-centered education	Patient and caregiver education	Control	Statistics and significance level
Variable	Variable levels	(percentage) frequency	(percentage) frequency	(percentage) frequency	(percentage) frequency	F* or X2 (P value)
Gender	female	19(61.3)	17(54.8)	15(48.4)	17(54.8)	1.042(0.791)
	Male	12(38.7)	14(45.2)	16(51.6)	14(45.2)	
Caregiver’s marital status	Single	13(41.9)	7(22.6)	5(16.1)	11(35.5)	6.263(0.100)
	Married	18(58.1)	24(77.4)	26(83.9)	20(64.5)	
Caregiver’s educational level	Elementary	4(12.9)	0(0)	3(9.7)	4(12.9)	13.504(0.321) *
	Middle School	3(9.7)	6(19.4)	8(25.8)	2(6.5)	
	Diploma	9(29.0)	13(41.9)	10(32.3)	8(25.8)	
	Associate Degree	7(22.6)	4(12.9)	3(9.7)	7(22.6)	
	Bachelor’s Degree and Higher	8(25.8)	8(25.8)	7(22.6)	10(32.3)	
Caregiver’s employment status	Housewife	13(41.9)	12(38.7)	13(41.9)	9(29.0)	14.40(0.446) *
	Student	3(9.7)	1(3.2)	2(6.5)	2(6.5)	
	Employee	4(12.9)	5(16.1)	5(16.1)	10(32.3)	
	Self-Employed Job	9(29.0)	13(41.9)	7(22.6)	11(35.5)	
	Retired	2(6.5)	0(0)	1(3.2)	0(0)	
Patient-caregiver family relationship	Spouse	9(29.0)	11(35.5)	13(41.9)	8(25.8)	8.713(0162) *
	sister or brother	1(3.2)	3(9.7)	4(12.9)	0(0)	
	child	21(67.7)	17(54.8)	14(45.2)	23(74.2)	
Place of residence (patient and caregiver)	City	27(87.1)	26(83.9)	23(74.2)	29(93.5)	4.662(0.198) *
	Village	4(12.9)	5(16.1)	8(25.8)	2(6.5)	
Caregiver’s income adequacy	Insufficient	8(25.8)	8(25.8)	6(19.4)	8(25.8)	0.852(0.991)
	Moderate	16(51.6)	17(54.8)	17(54.8)	17(54.8)	
	sufficient	7(22.6)	6(19.4)	8(25.8)	6(19.4)	
Smoking	No	27(87.1)	22(71.0)	22(71.0)	28(90.3)	6.162(0.104)
	Yes	4(12.9)	9(29.0)	9(29.0)	3(9.7)	
Caregiver’s diseases	No	22(71.0)	26(83.9)	23(74.2)	29(93.5)	2.067(0.559)
	Yes	9(29.0)	5(16.1)	8(25.8)	2(6.5)	
Receiving education on the therapeutic care regimen by caregiver	No	2(6.5)	2(6.5)	1(3.2)	1(3.2)	1.495(1.00)*
	Yes	29(93.5)	29(93.5)	30(96.8)	30(96.8)	
Getting help for care	No	6(19.4)	11(35.5)	6(19.4)	4(12.9)	5.066(0.167)
	Yes	25(80.6)	20(64.5)	25(80.6)	27(87.1)	

Chi-square or Fisher’s exact test is performed

* Fisher’s exact test is the reported statistic

Items without * are Chi-square

### Instruments

The data were collected using the patient and caregiver demographic forms and Novak and Guest care burden inventory (1989).

### Patient and caregiver demographic forms

Demographic forms for patients (age, gender) and caregivers (age, gender, marital status, educational level, employment status, caregiver-patient family relationship, income adequacy, place of residence of patient and caregiver, smoking, caregiver’s diseases and receiving education about treatment-care regimen and help for care) as well as disease information form for the patient (length of care, patient care hours per day and duration of kidney failure/month) were completed.

### Care burden inventory

The 24-item care burden inventory was developed by Novak and Guest (1989) to measure objective and subjective care burdens. This scale, consisting of five subscales of time-dependent (5 items), developmental (4 items), physical (4 items), social (5 items) and emotional (5 items) care burdens, assessed mental care burden with more emphasis [24]. The items were scored based on a 5-point Likert scale, ranging from completely false (0) to completely true [4]. Thus, the scores ranged from 24 to 120. Higher scores indicated higher care burden. The translation and cultural adaptation of the questionnaire was done by Abbasi et al. (2013). After examining and correcting the gap, a preliminary study was conducted on 40 individuals and its reliability was reported as 0.90 using the Cronbach’s alpha coefficient and 0.76–0.82 using the Cronbach’s alpha coefficient of subscales [25].

### Statistical analysis

The descriptive statistics (frequency, mean and standard deviation) was used to describe the research data. Shapiro-Wilk test was employed to examine data distribution. Demographic variables were compared between the four groups by one-way ANOVA and Chi-square test. The repeated measures ANOVA with modified LSD post hoc test was used for intragroup comparison of the main variables. One-way ANOVA with LSD post hoc test was used for intergroup comparison. Statistical analyses were performed by SPSS 19.0. The significance level was considered less than 0.05 in all cases.

## Results

**Fig. 1:**
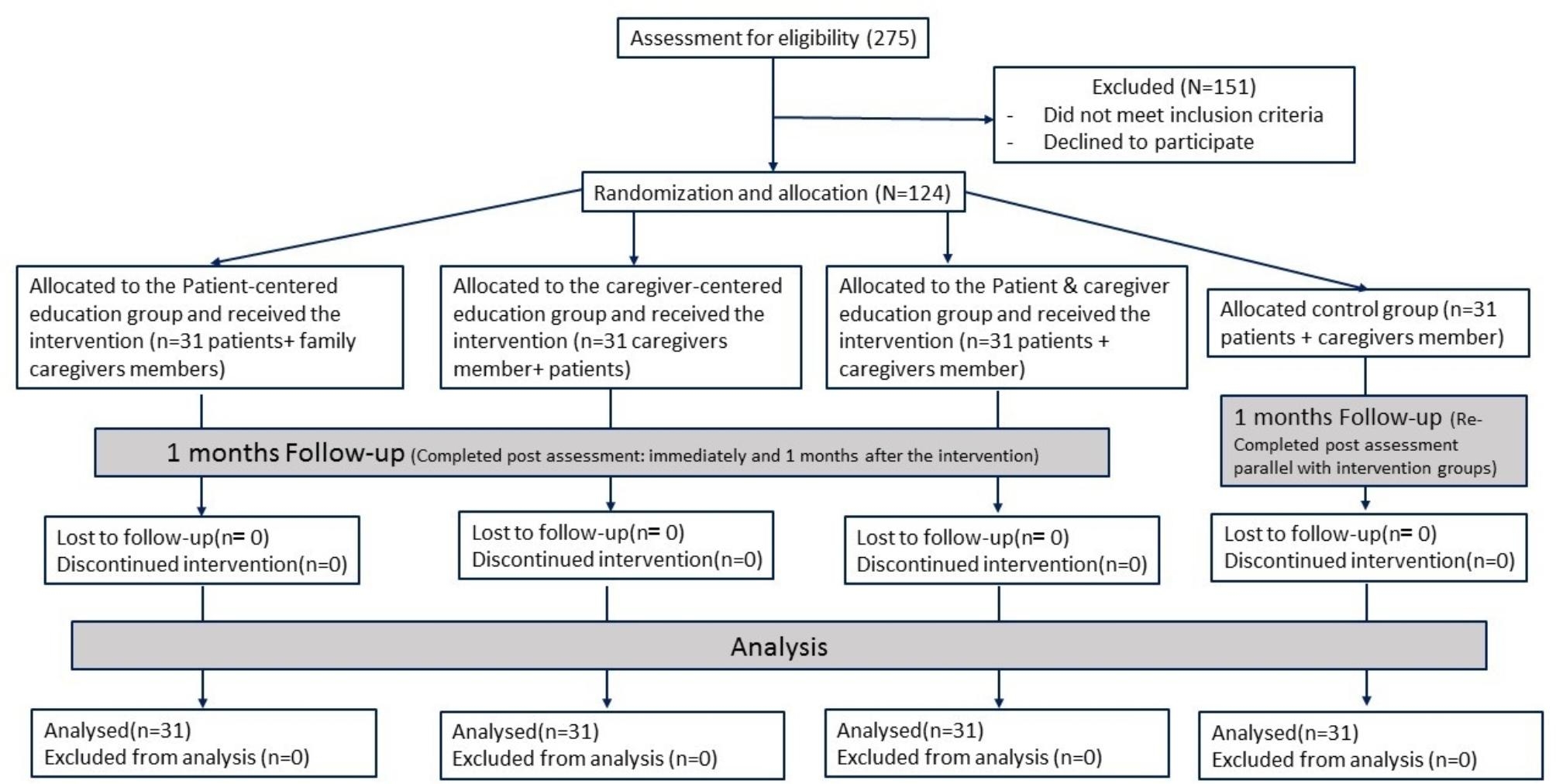
CONSORT flow chart of the study

This study was conducted on 124 patient-caregiver pairs, who were divided into four groups of patient-centered education, caregiver-centered education, Patient and caregiver education and control (n = 31 pairs per group). All the patients and caregivers participated in the study and the response rate was 100% (Fig. 1).

The analysis results showed there were no missing data. Out of the caregivers participating in the study, 68 (54.8%) were female and the rest were male. Among the patients, 86 (69.4%) were male and the other were female. The mean age of the caregivers in patient-centered, caregiver-centered, patient-caregiver and control groups were 39.84 ± 9.42,

38.12 ± 45.79, 38.10 ± 35.60 and 40.12 ± 16.42 years old, respectively. Also, the mean age of the patients in patient-centered, caregiver-centered, patient-caregiver and control groups were 55.39 ± 13.02, 54.14 ± 87.37, 54.14 ± 87.37 and 51.15 ± 81.03 years old, respectively. There was no statistically significant difference in other demographic variables between the four groups (Tables 1 and 2).

**Table 2:** Comparing mean and standard deviation of quantitative research variables between four groups

Group	Patient-centered education		Caregiver-centered education		Patient-caregiver education		Control		Statistics and significance level
Variable	mean	SD	mean	SD	mean	SD	mean	SD	F* or X2 (P value)
Patient’s age	55.39	13.02	54.87	14.37	54.87	14.37	51.81	15.03	0.521(0.669)*
Caregiver’s age	39.84	9.42	38.45	12.79	38.35	10.60	40.16	12.42	0.208 (0.891)
Duration of care/month	32.48	28.54	38.94	37.69	42.55	32.54	31.84	25.07	2.155(0.541)*
Patient care hours per day	5.23	2.62	6.13	2.70	5.58	1.86	5.58	1.71	0.840(0.475)
Duration of kidney failure/month	35.00	27.27	46.97	46.12	42.55	40.84	39.32	35.37	0.450(0.930)*
Time-dependent care burden pre-test score	6.26	4.01	7.68	5.53	7.00	5.53	5.71	4.68	0.924(0.431)
Developmental care burden pre-test score	11.03	4.52	11.65	4.22	12.23	2.74	11.03	4.52	0.503(0.681)
Physical care burden pre-test score	3.29	3.01	3.77	3.44	3.23	2.32	3.29	3.01	1.062(0.368)
Emotional care burden pre-test score	1.50	2.43	1.97	2.01	1.84	1.95	1.50	2.43	0.609(0.610)
Social care burden pretest score	3.90	3.35	4.13	3.93	3.58	2.75	3.90	3.35	0.434(0.729)
Total care burden pre-test score	25.87	12.54	29.19	13.42	27.87	9.64	25.87	12.54	0.497(0.685)

The items without * are analyzed by one-way ANOVA and reported statistic F.

The items with * are analyzed by Kruskal-Wallis test and reported statistic X2

The pre-test scores of total care burden in patient-centered education, caregiver-centered education, Patient and caregiver education and control groups were 25.87 ± 12.54, 29.19 ± 13.42, 27.87 ± 9.64 and 25.87 ± 12.54, respectively. Results of one-way ANOVA showed no statistical difference between the groups in the care burden and its dimensions in the pre-test (Table 2).

There was a statistically significant difference in the mean total care burden scores of the pre-test and post-test between the four groups (p < 0.001). Intragroup comparisons showed the total care burden decreased in patient-centered (-2.07 ± 3.02), caregiver-centered (-12.77 ± 6.42) and patient-caregiver (-13.19 ± 5.26) education groups. However, this reduction in caregiver-centered and Patient and caregiver education groups was significantly more than the patient-centered education group (p < 0.001) (Table 3).

**Table 3:** Intragroup comparison of mean care burden score and its domains

Variable	Patient-centered education		Caregiver-centered education		Patient and caregiver education		Control	
	Mean	SD	Mean	SD	Mean	SD	Mean	SD
Time-dependent care burden pre-test score (T1)	6.26	4.01	7.68	5.53	7.00	5.53	5.71	4.68
Time-dependent care burden post-test score (T2)	4.97	3.77	4.39	3.86	3.65	3.59	5.97	4.66
Time-dependent care burden follow-up score (T3)	5.13	3.75	4.58	3.92	3.71	3.60	6.10	4.66
F (P value)	29.321 (< 0.001)		62.176 (< 0.001)		61.247 (< 0.001)		3.958 (< 0.001)	
Pairwise comparison	T2 < T1*** T3 < T1*** T3 > T2*		T2 < T1*** T3 < T1*** T3 > T2*		T2 < T1*** T3 < T1*** T3/T2: NS		T2/T1: NS T3 > T1* T3/T2: NS	
Developmental care burden pre-test score	11.03	4.52	11.65	4.22	12.23	2.74	11.77	3.74
Developmental care burden post-test score	10.42	4.01	7.16	2.95	7.19	2.06	11.81	3.83
Developmental care burden follow-up score	10.68	4.00	7.68	3.23	7.74	2.24	12.03	3.67
F (P value)	3.176 (0.079)		86.910(< 0.001)		122.072 (< 0.001)		0.706 (0.425)	
Pairwise comparison	-		T2 < T1*** T3 < T1*** T3 > T2**		T2 < T1*** T3 < T1*** T3 > T2**		-	
Physical care burden pre-test score	3.29	3.01	3.77	3.44	3.23	2.32	2.52	2.25
Physical care burden post-test score	3.06	2.32	1.84	1.90	1.29	1.24	2.68	2.18
Physical care burden follow-up score	3.32	2.33	2.13	1.84	1.65	1.31	2.71	2.13
F (P value)	1.221 (0.287)		26.277 (< 0.001)		47.580 (< 0.001)		3.333 (0.057)	
Pairwise comparison	-		T2 < T1*** T3 < T1*** T3 > T2**		T2 < T1*** T3 < T1*** T3 > T2***		-	
Emotional care burden pre-test score	1.50	2.43	1.97	2.01	1.84	1.95	2.19	1.76
Emotional care burden post-test score	1.52	2.39	0.90	0.87	0.87	1.20	2.42	1.89
Emotional care burden follow-up score	1.68	2.45	1.10	1.04	0.94	1.09	2.48	1.95
F (P value)	1.241 (0.297)		13.572 (0.001)		15.907 (< 0.001)		7.336 (0.005)	
Pairwise comparison	-		T2 < T1*** T3 < T1** T3 > T2*		T2 < T1*** T3 < T1*** T3/T2:NS		T2 > T1* T3 > T1* T3/T2:NS	
Social care burden pre-test score	3.90	3.35	4.13	3.93	3.58	2.75	4.52	3.16
Social care burden post-test score	3.90	3.19	2.13	2.25	1.68	1.49	4.77	2.87
Social care burden follow-up score	4.032	3.18	2.45	2.26	2.03	1.64	4.97	3.02
F (P value)	0.795 (0.409)		63.397 (< 0.001)		28.367 (< 0.001)		7.081 (0.005)	
Pairwise comparison	-		T2 < T1*** T3 < T1*** T3 > T2**		T2 < T1*** T3 < T1*** T3 > T2**		T2/T1:NS T3 > T1** T3 > T2*	
Total care burden pre-test score	25.87	12.54	29.19	13.42	27.87	9.64	26.71	9.22
Total care burden post-test score	23.87	11.31	16.42	8.11	14.68	6.37	27.65	9.55
Total care burden follow-up score	24.71	11.37	17.61	8.53	15.71	6.15	28.10	9.45
F (P value)	9.766 (< 0.001)		109.447 (< 0.001)		167.125 (< 0.001)		10.996 (0.001)	
Pairwise comparison	T2 < T1** T3/T1: NS T3 > T2***		T2 < T1*** T3 < T1*** T3 > T2***		T2 < T1*** T3 < T1*** T3 > T2***		T2 > T1* T3 > T1*** T3 > T2***	

Repeated measures ANOVA is performed

*: P-value < 0.05; **:P value < 0.01; ***P-value < 0.001

T1: pretest; T2: posttest; T3: Follow up; Ns: non-significant

Intragroup comparisons showed an incremental change from the pre-test to follow-up in the control group (1.58 ± 2.22). However, this change was decremental in caregiver-centered and Patient and caregiver education groups (Table 3).

Intergroup comparisons showed no statistically significant difference between caregiver-centered and Patient and caregiver education groups in the mean total care burden scores of the pre-test and post-test (p = 0.719) (Table 4).

**Table 4:** Comparing mean changes in the total care burden between the four groups among the caregivers participating in the research

Group		T2-T1	T3-T1	T3_T2
Patient-centered education	Mean	-2.07	-1.10	0.97
	SD	3.02	3.20	0.60
Caregiver-centered education	Mean	-12.77	-11.26	1.52
	SD	6.42	6.39	0.85
Patient and caregiver education	Mean	-13.19	-11.81	1.39
	SD	5.26	5.47	0.95
Control	Mean	0.94	1.58	0.65
	SD	2.29	2.22	0.71
Comparing the four groups	F statistic	78.133	68.861	7.863
	Significance level	< 0.001	< 0.001	< 0.001
Comparing patient-centered and caregiver-centered education	95% confidence interval for mean difference	< 0.001 8.389; 13.026	< 0.001 7.803; 12.513	0.007 − 0.946; − 0.150
Comparing patient-centered and patient and caregiver education	95% confidence interval for mean difference	< 0.001 8.808; 13.446	< 0.001 8.351; 13.062	0.039 − 0.817; − 0.021
Comparing patient-centered education and control	95% confidence interval for mean difference	0.012 -5.321; − 0.683	0.026 -5.036; − 0.325	0.111 − 0.075; 0.721
Comparing caregiver-centered and patient and caregiver education	95% confidence interval for mean difference	0.719 -1.880; 2.719	0.643 -1.788; 2.884	0.522 − 0.269; 0.527
Comparing caregiver-centered education and control	95% confidence interval for mean difference	< 0.001 -16.009; -11.410	< 0.001 -15.175; -10.503	< 0.001 0.473; 1.269
Comparing Patient and caregiver education and control	95% confidence interval for mean difference	< 0.001 11.829; 16.429	< 0.001 -15.723; -11.051	< 0.001 0.344; 1.140

The performed test: One-way ANOVA with LSD post hoc test

There was an incremental change from the pre-test to post-test in the control group (0.94 ± 2.29). However, this change was detrimental in the other three groups (Table 3). There was a statistically significant difference between the control and the other three groups (p < 0.001) (Table 4).

There was a significant difference in the mean care burden of follow-up and pre-test between the four groups. The pair-wise comparison showed the total care burden decreased in caregiver-centered (-11.26 ± 6.39) and patient-caregiver (-11.81 ± 5.47) education groups and increased in the patient-education group (-1.10 ± 3.20), and the difference between the patient-education group and the other two mentioned groups was statistically significant (p < 0.001) (Table 4).

There was no statistically significant difference between the caregiver-centered and Patient and caregiver education groups in terms of the mean total care burden scores of the pre-test and follow-up (p = 0.643) (Table 4).

There was a statistically significant difference between the control group and these two groups (p < 0.001). However, there was no statistically significant difference between the control and patient-centered education group (p > 0.05) (Table 4).

**Table 5:** Comparing mean changes in developmental and physical care burdens between four groups of caregivers

		Developmental care burden			Physical care burden		
Group		T2-T1	T3-T1	T3_T2	T2-T1	T3-T1	T3_T2
Patient-centered education	Mean	-0.61	-0.35	0.26	-0.23	0.03	0.26
	SD	1.56	1.68	0.51	1.18	1.20	0.44
Caregiver-centered education	Mean	-4.48	-3.97	0.52	-1.94	-1.65	0.29
	SD	2.34	2.61	0.77	1.82	2.04	0.46
Patient-caregiver education	Mean	-5.03	-4.48	0.55	-1.94	-1.58	0.35
	SD	2.24	2.43	0.81	1.39	1.41	0.49
Control	Mean	0.03	0.26	0.23	0.16	0.19	0.03
	SD	1.52	1.63	0.50	0.45	0.54	0.31
Comparing the four groups	F statistic	55.129	40.233	2.005	22.325	15.698	3.271
	Significance level	< 0.001	< 0.001	0.117	< 0.001	< 0.001	0.024
Comparing patient-centered and caregiver-centered education	Significance level	< 0.001	< 0.001	-	< 0.001	< 0.001	0769
	95% confidence interval for mean difference	2.890; 4.852	2.538; 4.687	-	1.052; 2.368	0.971; 2.383	-0.249; 0.185
Comparing patient-centered and patient and caregiver education	Significance level	< 0.001	< 0.001	-	< 0.001	< 0.001	0.379
	95% confidence interval for mean difference	3.438; 5.401	3.055; 5.203	-	1.052; 2.368	0.907; 2.319	-0.314; 0.120
Comparing patient-centered education and control	Significance level	0.169	0.261	-	0.246	0.652	0.042
	95% confidence interval for mean difference	-1.627; 0.336	-1.687; 0.462	-	-1.045; 0.271	-0.867; 0.545	0.009; 0.443
Comparing caregiver-centered and patient and caregiver education	Significance level	0.271	0.343	-	1.00	0.857	0.558
	95% confidence interval for mean difference	-0.433; 1.530	− 0.558; 1.591	-	-0.658; 0.658	-0.770; 0.641	-0.282; 0.153
Comparing caregiver-centered education and control	Significance level	< 0.001	< 0.001	-	< 0.001	< 0.001	0.020
	95% confidence interval for mean difference	-5.498; -3.535	-5.300; -3.151	-	-2.755; -1.439	-2.545; -1.133	0.041; 0.475
Comparing Patient and caregiver education and control	Significance level	< 0.001	< 0.001	-	< 0.001	< 0.001	0.004
	95% confidence interval for mean difference	-6.046; -4.083	-5.816; -3.667	-	-2.755; -1.439	-2.480; -1.068	0.105; 0.540

The performed test: One-way ANOVA with LSD post hoc test

There was a statistically significant difference in the mean total care burden scores of the post-test and follow-up between the four groups. Intragroup comparisons showed all the groups had an increase in the mean score in the follow-up compared to the post-test. Intergroup comparisons showed the increase in the control group (-1.10 ± 3.20) and patient-centered education group (0.97 ± 0.60) was significantly less than the caregiver-centered (1.52 ± 0.85) and patient-caregiver (1.39 ± 0.95) education groups (p < 0.001). No statistically significant difference was observed between the caregiver-centered and Patient and caregiver education groups (p = 0.522). Moreover, there was no statistically significant difference between the patient-centered education and control groups (p = 0.111).

Tables 4 and 6 present intragroup and intergroup comparisons of care burden domains, respectively.

The result showed that there was statistically significant difference between the patient education and caregiver education groups and between the patient education and patient and caregiver education groups in physical care burdens in the post-test (Table 5). But There was no statistically significant difference between the two groups of education to caregiver and education to patient and caregiver in physical care burdens in the post-test (Table 5).

The result showed that there was statistically significant difference between the control groups and the patient education and caregiver education groups and between the patient education and patient and caregiver education groups in the post-test and follow-up phases in total Developmental care burden scores in the post-test and follow-up phases (Table 5).

The result showed that there was statistically significant difference between the control groups and the patient education and caregiver education groups and between the patient education and patient and caregiver education groups in the post-test and follow-up phases in total emotional, and social care burden scores in the post-test and follow-up phases (Table 6).

**Table 6:** Comparing mean changes in social and emotional care burdens between four groups of caregivers

		Emotional care burden			Social care burden			
Group		T2-T1	T3-T1	T3-T2	T2-T1	T3-T1	T3-T2	
Patient-centered education	Mean	0.01	0.13	0.16	0.01	0.13	0.13	
	SD	0.53	0.68	0.37	0.77	0.76	0.34	
Caregiver-centered education	Mean	-1.06	-0.87	0.19	-2.00	-1.68	0.32	
	SD	1.41	1.50	0.40	1.81	1.89	0.54	
Patient and caregiver education	Mean	-0.97	-0.90	0.06	-1.90	-1.55	0.35	
	SD	1.05	1.16	0.36	1.74	1.82	0.61	
Control	Mean	0.23	0.29	0.06	0.26	0.45	0.19	
	SD	0.50	0.53	0.25	0.77	0.77	0.40	
Comparing the four groups	F statistic	14.746	11.472	1.114	24.105	18.900	1.499	
	Significance level	< 0.001	< 0.001	0.346	< 0.001	< 0.001	0.218	
Comparing patient-centered and caregiver-centered education	Significance level	< 0.001	< 0.001	-	< 0.001	< 0.001	-	
	95% confidence interval for mean difference	0.581; 1.548	0.474; 1.534	-	1.312; 2.688	1.093; 2.520	-	
Comparing patient-centered and patient and caregiver education	Significance level	< 0.001	< 0.001	-	< 0.001	< 0.001	-	
	95% confidence interval for mean difference	0.484; 1.451	0.506; 1.567	-	1.215; 2.591	0.964; 2.391	-	
Comparing patient-centered education and control	Significance level	0.357	0.559	-	0.459	0.373	-	
	95% confidence interval for mean difference	-0.709; 0.258	-0.687; 0.373	-	-0.946; 0.430	-1.036; 0.391	-	
Comparing caregiver-centered and patient and caregiver education	Significance level	0.690	0.904	-	0.781	0.721	-	
	95% confidence interval for mean difference	-0.576; 0.383	-0.494; 0.558	-	-0.785; 0.591	-0.843; 0.585	-	
Comparing caregiver-centered education and control	Significance level	< 0.001	< 0.001	-	< 0.001	< 0.001	-	
	95% confidence interval for mean difference	-1.770; -0.811	-1.687; -0.636	-	-2.946; -1.570	-2.843; -1.415	-	
Comparing Patient and caregiver education and control	Significance level	< 0.001	< 0.001	-	< 0.001	< 0.001	-	
	95% confidence interval for mean difference	-1.673; -0.714	-1.719; -0.668	-	-2.849; -1.473	-2.714; -1.286	-	

The performed test: One-way ANOVA with LSD post hoc test

## Discussion

This study was conducted to compare the effect of teaching health-promoting behaviors through caregiver- and patient-centered approaches on the care burden of family caregivers of patients undergoing hemodialysis. The intragroup results showed caregiver-centered, patient-centered and Patient and caregiver education reduced care burden immediately and one month after teaching compared to before teaching. However, there was no change in the care burden of the control group over time. It should be noted that the care burden in the patient-centered education group decreased only in time-dependent domain and mean total care burden score. This difference was not observed in other domains. Moreover, most of the domains and total care burden reduced in the follow-up compared to the post-test. The intergroup results showed care burden in caregiver-centered and Patient and caregiver education groups decreased more than the patient-centered education group. The mean change in caregiver-centered and Patient and caregiver education groups was more than the patient-centered education and control groups. The results revealed teaching the caregiver had a greater effect on the care burden and its domains. There was a statistically significant difference in the total care burden between the patient-centered education and control groups, but this difference was not significant in care burden domains, indicating lack of effect of patient-centered education on caregivers’ care burden.

The studies conducted on the effect of educational interventions on care burden have mostly focused on caregivers. Family-centered and paired education has not separated and compared the effect of teaching the main person and other family members. Studies examining only caregiver-centered education have reported that educational intervention could reduce care burden and its domains. Farahani et al. (2016), which was in line with the present study in terms of the effect of caregiver-centered education on the care burden [2]. Research on other diseases such as caregivers of the elderly with cancer [26], family caregivers of patients with coronary artery surgery [27] and patients with stroke [16] have demonstrated caregiver-centered education could reduce caregivers’ care burden, which was in line with the present work.

Some studies have investigated family-centered and paired education (patient and caregiver education). Sotoudeh et al. (2019), Rabiei et al. (2020) and Masoudi et al. (2020) showed family-centered educational intervention could reduce the caregivers’ care burden [6, 28, 29], which was consistent with the present study. Badr et al. (2015) found that teaching stroke patients and their caregivers reduced the caregivers’ care burden [30]. Although this study was not conducted on hemodialysis patients, since it showed performing intervention on patients and caregivers could reduce the care burden, it was in line with the present study. However, this study and those that have examined family-centered and paired education have not separated and compared patient-centered and caregiver-centered education. Therefore, it is not possible to find out whether teaching both the patient and caregiver is more effective than teaching the caregiver alone. In this sense, it could not be compared with the present study.

Liljeroos et al. (2017) and Agren et al. (2015) found that the educational intervention had no effect on the care burden [4, 31], which was not in line with our finding. The reason for this inconsistency could be attributed to the time of measuring the education outcome. Liljeroos et al. measured the care burden 24 months after the intervention, while Agren et al. assessed it 3 and 12 months after the intervention. In the present study, the outcome was measured immediately and one month after the intervention. Considering the increased care burden in the follow-up compared to the post-test, in case of continuing the measurement, the care burden could most likely reach the pre-test or even more. In the studies by Liljeroos et al. and Agren et al., the care burden was not measured immediately after teaching. Thus, the initial effect of teaching could not be evaluated.

Hekmatpou et al. (2019) studied patients with stroke and showed patient care education reduced caregivers’ care burden and its domains [16]. The obtained result of total care burden was consistent with the present research. However, the obtained result of care burden domains was not in line with the present study. In our investigation, the intergroup comparison showed a difference only in time-dependent care burden between the control and other groups, which led to a significant difference in the total care burden. Considering this finding and those of the other two groups showed the poor effect of patient-centered education on reducing the care burden. However, Hekmatpou et al. found a significant reduction in care burden after the intervention. This inconsistency could be due to the research tool and population. Hekmatpou et al. used Zarit burden of care questionnaire for measuring care burden from socioeconomic, physical and mental aspects. However, the instrument used in the present study measured domains such as time-dependent, developmental, emotional and physical care burdens. Difference in the research population could be more likely the reason for inconsistency in the effectiveness of the intervention. Dialysis patients were included in the present study, while Hekmatpou et al. examined stroke patients. In the present study, care burden was low because most of the dialysis patients could usually do their personal tasks and often go to dialysis alone, but patients with stroke need more care. Hekmatpou et al. reported that care burden was high before the intervention, and increasing patients’ awareness and, consequently, their cooperation could reduce caregivers’ care burden. Moreover, they found that intervention decreased caregivers’ care burden from high to moderate level and the intervention could not bring the mean care burden score below the moderate level. However, in the present study, the mean care burden score before the intervention was lower than the moderate level and the intervention slightly reduced it. Patient-centered or Patient and caregiver education has an optimal effect on reducing caregivers’ care burden if this is used for some diseases that greatly reduce patients’ ability or in cases that care burden is high.

The results revealed providing training to caregivers has a greater effect on the care burden. Teaching caregivers how to manage patient-specific problems could improve caregiver well-being [32]. If caregivers know what health-promoting behaviors are and behave accordingly, it will make patients perform the desired behavior. This lifestyle change could help patients’ independence and improve their quality of life. It seems that caregivers who receive training and know their patients’ health-promoting behaviors recognize disease symptoms when they occur and feel confident in what to do if they worsen. Thus, they experience less burden and are more confident in their care. When disease symptoms manifest, the patient often consults their caregivers. If caregivers do not have the knowledge of how to support the patient when symptoms manifest or their lifestyle changes, it may be difficult for the patient to follow self-care instructions [4]. This could act as a vicious cycle and affect the caregiver’s care burden due to poor adherence or non-adherence. Therefore, considering the undeniable role of family caregivers of hemodialysis patients in improving the effectiveness of the provided care services, it is recommended that healthcare providers, especially nurses, pay attention to the needs of caregivers and patients [33]. Healthcare providers should first evaluate caregivers’ educational needs to reduce the care burden and present their educational interventions and plans accordingly. For this purpose, they could take steps to improve caregivers’ health using educational methods such as teach-back method.

Considering that the effect of teaching on caregiver-centered and Patient and caregiver education groups was almost similar, the educational intervention results did not confirm the paired adaptation model among the dialysis patients participating in the present study and their caregivers [17]. Probably, participation of patients and caregivers in accepting responsibilities, degree of adaptation to stress and participation in adaptation to stress were different. The paired adaptation model could be adjusted to the levels of patient and caregiver outcomes, including caregivers’ care burden as well as type of disease. However, these assumptions require further studies. Since no study has ever examined the effect of education on patients and caregivers in four groups, conducting further studies in this field on dialysis patients and their caregivers as well as other chronic diseases could help obtain more accurate results.

The results showed in groups that the educational intervention reduced the care burden, in most cases, the care burden increased in the follow-up compared to immediately after the intervention. Except for time-dependent and emotional care burdens in the Patient and caregiver education group which were not significantly different between the post-test and follow-up, the results showed after implementing the intervention, its effect would decrease over time. Deyhoul et al. (2019) indicated family-centered education was effective in reducing the care burden of stroke patients’ caregivers, and the care burden decreased in the follow-up compared to the post-test [34], which was consistent with the present study. These results revealed teaching should be repeated to maintain the effect of teaching on the care burden. Teaching repetition intervals should be planned based on the target population and their monitoring. Different teaching methods should be compared to determine which one has more stable effects. Thus, further studies are required in this field.

## Conclusion

The intervention reduced the care burden and teaching health-promoting behaviors through caregiver-centered approach could reduce the care burden more than patient-centered approach. Therefore, it could be used as a support method to reduce caregivers’ care burden. It is necessary for healthcare providers, especially nurses, to provide both patient-centered and caregiver-centered education and include educational programs for caregivers in nursing education. Using other teaching approaches in addition to teach-back method and comparing them with this type of teaching method could determine the most effective method for dealing with the care burden resulting from the care of hemodialysis patients. Therefore, it is recommended to conduct further research in this field.

## Data Availability

All data is available in the text of the manuscript, Tables, and Additional files.
